# Effect of Nickel Ions on the Physiological and Transcriptional Responses to Carbon and Nitrogen Metabolism in Tomato Roots under Low Nitrogen Levels

**DOI:** 10.3390/ijms231911398

**Published:** 2022-09-27

**Authors:** Kun Zhang, Shuhao Li, Yang Xu, Yuqi Zhou, Shengxiang Ran, Huanhuan Zhao, Weiqun Huang, Ru Xu, Fenglin Zhong

**Affiliations:** 1 College of Horticulture, Fujian Agriculture and Forestry University, Fuzhou 350002, China; 2 Fujian Seed Station, Fuzhou 350001, China

**Keywords:** nickel, tomato root, RNA-seq, carbon and nitrogen metabolism, glycolytic pathway-tricarboxylic acid cycle, low nitrogen, biosynthesis of amino acids

## Abstract

Nickel (Ni) is an essential trace element for plant growth and a component of the plant body that has many different functions in plants. Although it has been confirmed that nickel ions (Ni^2+^) havea certain regulatory effect on nitrogen (N) metabolism, there are not enough data to prove whether exogenous Ni^2+^ can increase the carbon (C) and N metabolism in the roots of tomato seedlingsunder low-nitrogen (LN) conditions. Therefore, through the present experiment, we revealed the key mechanism of Ni^2+^-mediated tomato root tolerance to LN levels. Tomato plants were cultured at two different N levels (7.66 and 0.383 mmol L^−1^) and two different Ni^2+^ levels (0 and 0.1 mg L^−1^ NiSO_4_ 6H_2_O) under hydroponic conditions. After nine days, we collected roots for physiological, biochemical, and transcriptome sequencing analyses and found that the activities of N assimilation-related enzymes decreased at LN levels. In contrast, Ni^2+^ significantly increased the activities of N assimilation-related enzymes and increased the contents of nitrate (NO_3_^−^), ammonium (NH_4_^+^), and total amino acids. Through root transcriptomic analysis, 3738 differentially expressed genes (DEGs) were identified. DEGs related to C and N metabolism were downregulated after LN application. However, after Ni^2+^ treatment, *PK*, *PDHB*, *GAPDH*, *NR*, *NiR*, *GS*, *GOGAT*, and other DEGs related to C and N metabolism were significantly upregulated. In conclusion, our results suggest that Ni^2+^ can regulate the C and N metabolism pathways in tomato roots to alleviate the impact of LN levels.

## 1. Introduction

Nitrogen (N) is an essential mineral nutrient element in plants; it is an important component of proteins, nucleic acids, phospholipids, and certain growth hormones. It also accounts for 40–50% of the total final crop yield [1,2,3]. Therefore, the application of N fertilizer in agricultural production often increases crop yield. In actual production, vegetable farmers often overapply N fertilizer, far exceeding the needs of the crop, to increase economic efficiency [4,5]. However, instead of improving crop yield and quality, excessive N fertilizer inputs have reduced efficiency and increased N fertilizer losses, causing serious environmental problems [6].

Nickel (Ni) is considered an important plant micronutrient due to its different biological functions [7]. Plants grown in Ni-deficient nutrient solutions may show significant Ni deficiency symptoms such as the “mouse-ear or little-leaf” (ME–LL) found in pecan [8,9]. In addition, Ni is the only known urease activator that activates urease to then hydrolyze urea to ammonia and carbon dioxide [10], thereby avoiding the toxic effects of urea accumulation. Further, urea accumulation can lead to severe limitations in the supply of ammonia for the synthesis of certain amino acids and various types of proteins, which in turn appears to cause other disruptions in N metabolism (in particular, the disruption of glutamine synthase [11,12]), which is ultimately detrimental to plant growth. On the other hand, Ni is also a component of other enzymes responsible for nitrate reduction and is therefore involved in N assimilation in plants [13]. A study on cowpea found that Ni and urease were involved in N metabolism during the reproductive growth phase of legumes [14]. Other studies have shown that the application of nickel at low concentrations can increase the level of plant N metabolism and have a beneficial effect on plant growth and development [15,16,17]. Through a previous experiment, we showed that exogenous nickel ions (Ni^2+^) could regulate genes involved in the EMP pathway–TCA cycle, amino acid biosynthesis, and N metabolism in tomato leaves [18]. As a major site of N uptake, the physiological characteristics of the tomato roots system directly influence the growth and development of tomatoes. However, it is not clear how the root system responds to exogenous Ni^2+^ regulation under low-nitrogen (LN) conditions. Thus, physiological, biochemical, and transcriptomic analyses were used in this study to analyzethe changes to the carbon (C) and N metabolism in the roots of tomato seedlings following Ni^2+^ applications under LN conditions.

## 2. Results

### 2.1. Carbohydrate Content

**Figure 1:**
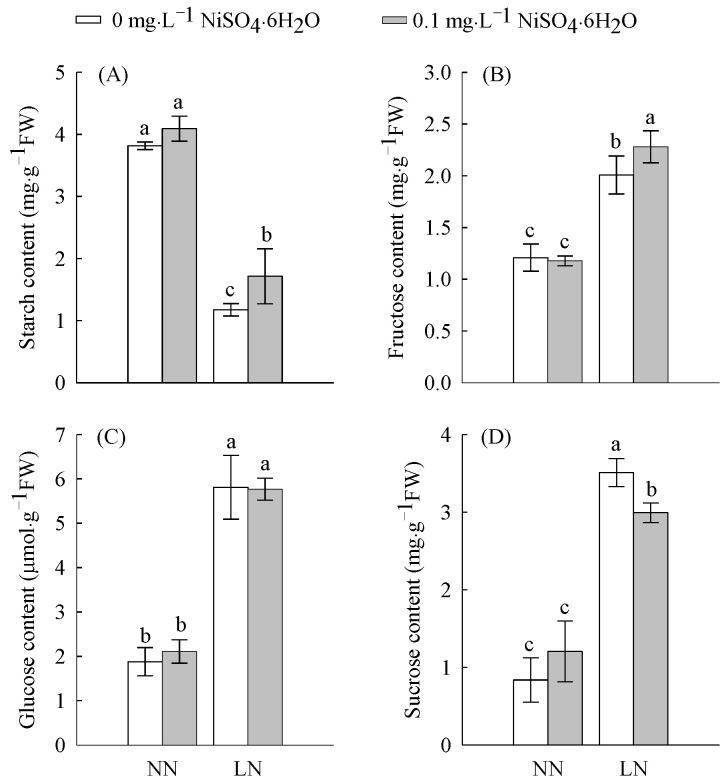
The contents of starch (**A**), fructose (**B**), glucose (**C**), and sucrose (**D**) in tomato roots treated with LN and Ni^2+^. Data are presented as the mean ± SD of three independent biological replicates. Different lowercase letters in the same column indicate significant differences at the 0.05 level among treatments.

The starch content at LN levels was significantly decreased by 54% compared with normal nitrogen (NN) levels, while fructose was significantly increased by 40%, glucose by 68%, and sucrose by 76% (Figure 1A–D). These results show that starch is decomposed in large quantities under LN levels and that the three soluble sugars—fructose, glucose, and sucrose—are synthesized in large quantities to resist adversity. The starch and fructose contents increased significantly by 46 and 13%, respectively, under LN conditions after Ni^2+^ addition.

### 2.2. Enzymatic Activities and N Content

**Figure 2:**
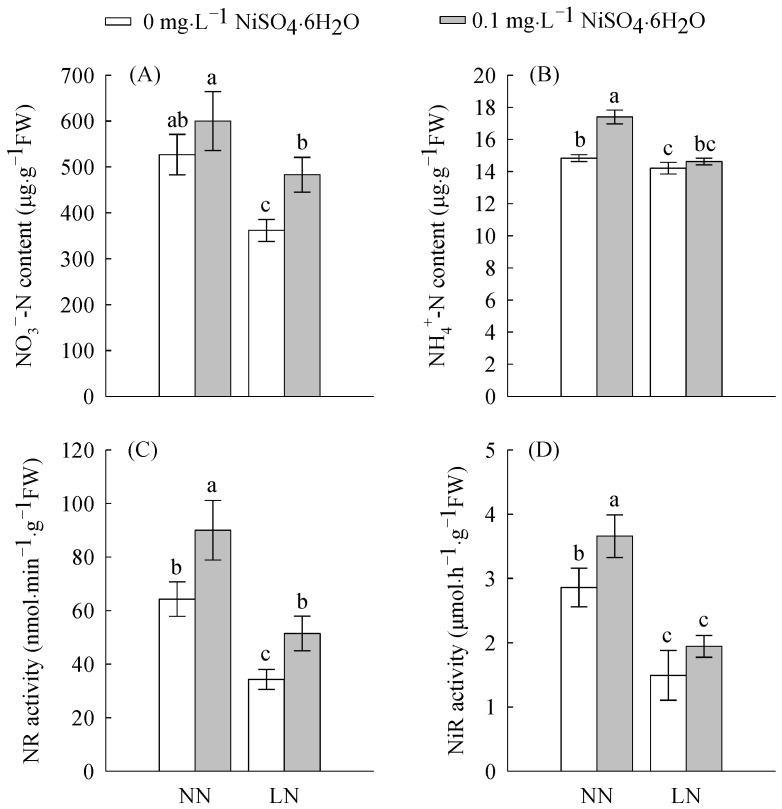
The NO_3_^−^ content (**A**), NH_4_^+^ content (**B**), NR (**C**), and NiR (**D**) in tomato roots treated with LN and Ni^2+^. Data are presented as the mean ± SD of three independent biological replicates. Different lowercase letters in the same column indicate significant differences at the 0.05 level among treatments.

**Figure 3:**
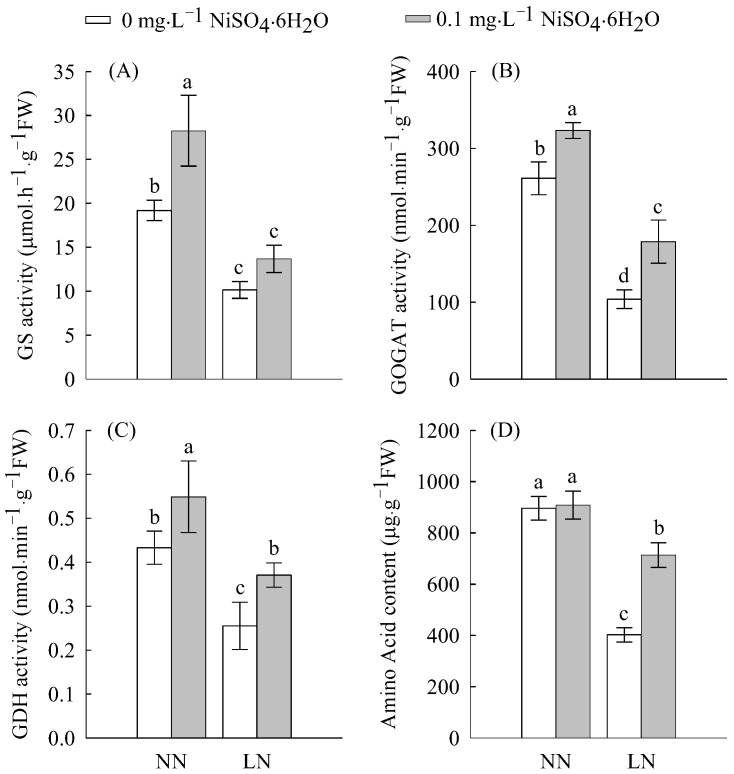
The GS (**A**), GOGAT (**B**), GDH (**C**), and total amino acid content (**D**) in tomato roots treated with LN and Ni^2+^. Data are presented as the mean ± SD of three independent biological replicates. Different lowercase letters in the same column indicate significant differences at the 0.05 level among treatments.

As shown in Figure 2 and Figure 3, Ni^2+^ treatment increased the enzymatic activities of nitrate reductase (NR), nitrite reductase (NiR), glutamine synthetase (GS), glutamate synthetase (GOGAT), glutamate dehydrogenase (GDH) and the contents of nitrate nitrogen (NO_3_^−^), ammonium nitrogen (NH_4_^+^), and the total amino acids. Under LN treatment, all enzymatic activities and contents of N compounds decreased in comparisonwith NN; however, LN significantly inhibited NR (47%), NiR (47%), GS (47%), GDH (41%), and GOGAT (60%) activities. This indicates that LN levels inhibit N assimilation, thereby inhibiting the formation of nitrogen-containing compounds. However, after Ni^2+^ treatment, the contents of NR, NiR, GS, GOGAT, GDH, NO_3_^−^, NH_4_^+^, and the total amino acids all increased under LN conditions. Under LN conditions, the addition of Ni^2+^ significantly increased the activities of NR, GDH, and GOGAT by 80, 45 and 72%, respectively. This indicates that Ni^2+^ can regulate N metabolism.

### 2.3. Analysis of Differentially Expressed Genes (DEGs)

**Figure 4:**
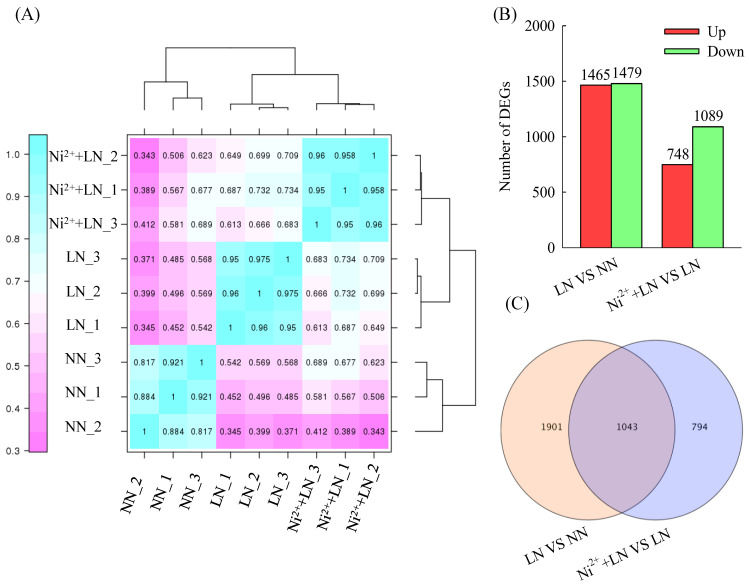
Statistics of the transcriptome in the tomato roots. Pearson’s correlation analysis (**A**), number of DEGs (**B**), and Venndiagram (**C**).

We found that each mRNA library contained a total of 40.68 to 54.42 million clean reads with a Q30 percentage ≥91% (Table 1). We obtained approximately 41.27 million, 50.11 million and 41.95 million clean reads from the NN group, LN group, and Ni^2+^ + LN group, respectively. We retained a large proportion (approximately 89.42, 89.47 and 90.45%) of clean reads (approximately 36.89 million, 44.91 million and 37.94 million for the NN group, LN group, and Ni^2+^ + LN group, respectively) for assembly and downstream analysis. In addition, we obtained approximately 36.08 million, 44.15 million, and 37.24 million reads from the NN group, LN group, and Ni^2+^ + LN group, respectively, which were aligned to the unique position of the reference genome. The percentage of reads aligned to the unique position of the reference genome in the clean reads was approximately 87.42, 87.96 and 88.78%, for the NN group, LN group, and Ni^2+^ + LN group, respectively. We identified 3738 DEGs using these treatments by analyzingthe full variation in DEGs across different databases. Overall, 2944 and 1837 genes were differentially expressed between the LN vs. NN groups and the Ni^2+^ + LN vs. LN groups, respectively. Among these DEGs, 1465 and 748 genes were upregulated, and 179 and 1089 were downregulated (Figure 4A–C and Table S1).

### 2.4. Functional Classification of DEGs and Validation by Quantitative Real-Time PCR (RT-qPCR)

**Figure 5:**
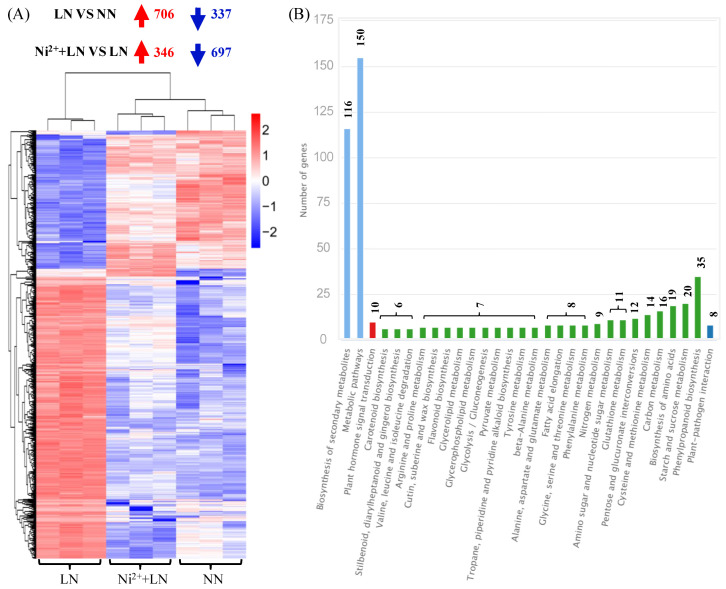
Statistics for 1043 DEGs in the intersection of two comparative groups. Hierarchical clustering (**A**) and KEGG classification (**B**).

The Venn diagram showed the existence of 1043 co-DEGs between the two comparison groups (Figure 4C and Table S2), which can be regarded as the central DEGs for the exploration of the corresponding co-acting mechanism. These 1043 common DEGs were subjected to hierarchical clustering analysis, in which 706 or 346 genes were upregulated and 337 or 697 genes were downregulated in the LN vs. NN group and the Ni^2+^ + LN vs. LN group, respectively (Figure 5A and Table S2). We performed a KEGG classification analysis on these common DEGs, and the results showed the main and other metabolic pathways involved (Figure 5B and Table S3). We used primers related to C metabolism (*Solyc08g079080.4*, *Solyc11g007690.2*) and N metabolism (*Solyc03g083440.3, Solyc04g014510.3*, *Solyc09g010970.3*, *Solyc10g050890.2*, and *Solyc11g013810.2*). We verified the authenticity of the RNA-seq data and the relative expression levels of the eight DEGs of interest by RT-qPCR. We found that the RT-qPCR results were consistent with the RNA-seq expression profile (Table 2), thus demonstrating the authenticity of the RNA-seq data.

### 2.5. Metabolic Regulation of Enzymes Encoded by DEGs

**Figure 6:**
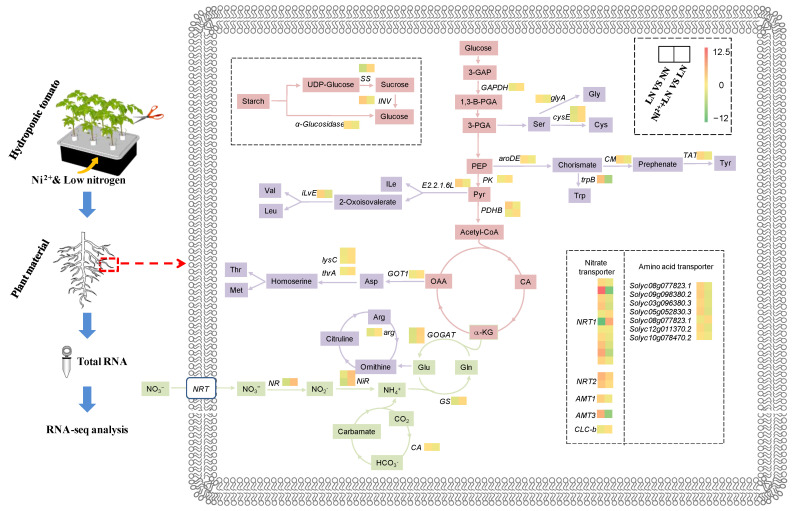
A simplified transcriptional map based on associated transcriptional pathways in tomato roots in response to Ni^2+^ and LN levels. Each square represents DEGs, and the color indicates the log_2_(fold change) of the DEGs (|log_2_(fold change)| ≥ 1 and *p*-adjusted value < 0.01; red indicates upregulation, and green indicates downregulation).

The common DEGs were significantly enriched in C and N metabolic pathways. LN application inhibited the expression levels of pyruvate kinase (*PK*), pyruvate dehydrogenase E1 component beta subunit (*PDHB*), glyceraldehyde-3-phosphate dehydrogenase (*GAPDH*), nitrate reductase (*NR*), ferredoxin-nitrite reductase (*NiR*), glutamine synthetase (*GS*), and glutamate synthase (*GOGAT*) (Figure 6 and Table S1). However, in the Ni^2+^ treatment (Ni^2+^ + LN), the expression levels of these genes were all increased, and *NR* and *NiR* were significantly increased by 4.3 and 3.6 fold, respectively. The results for NR, NiR, GS, and GOGAT in the N assimilation pathway were essentiallyconsistent with the physiological detection results of the enzymes.

## 3. Discussion

As an extremely common limiting factor for plant growth and development, N plays a vital role in various metabolic processes. Efficient N supply and utilization result in extensive physiological and biochemical changes in plants [19], which, in turn, reduce plant growth. Correspondingly, plants can respond to changes in N availability through changes in morphological, physiological, and biochemical pathways [20,21]. Under LN levels, the activities of key N-assimilation enzymes such as NR/NiR and GOGAT/GS (Figure 2 and Figure 3) and their related genes (*Solyc04g014510.3*, *Solyc03g083440.3*, *Solyc08g044270.3*, *Solyc11g013810.2*, *Solyc01g108630.3*, and *Solyc10g050890.2*) were significantly reduced; they alsodisrupted the transcript levels of amino acid biosynthesis-related genes (Figure 6 and Table S2), thereby reducing the accumulation of free amino acids (Figure 3), and therefore indicating that N assimilation is affected by inhibition. Similarly, a dramatic downregulation of N assimilation-related DEGs were found in apples and rice [22,23] during N starvation. The downregulation of these DEGs may be a feedback response to N starvation; however, further studies are needed to evaluate this hypothesis. Exogenous Ni^2+^ increased the concentration of NO_3_^−^ in roots under LN conditions, thereby promoting the activities of NR and NiR, and their related gene expression levels (Figure 3 and Table S2), which in turn promoted the reduction of NO_3_^−^. GS/GOGAT is crucial for regulating the N cycle and NH_4_^+^ assimilation in response to adverse environments [24]. Exogenous Ni^2+^ induces GS/GOGAT activity under LN levels and mediates the transcription level of its related coding genes, which may help promote NH_4_^+^ reassimilation under low-N conditions to improve plant tolerance to N deficiency [25]. As an important form and the main transport form of N assimilation in plants, free amino acids can reflect the supply capacity of N assimilates [26]. The supply of Ni^2+^, under LN conditions, readjusted the transcription levels of genes encoding amino acid synthesis-related enzymes in tomato roots (Figure 6 and Table S2) in order to maintain the normal generation of amino acids and ensure an adequate supply of N assimilates. At the same time, various amino acids must be transported to specific sites by intracellular amino acid transporters to perform their respective functions [27]. In this study, LN supply upregulated amino acid transporter genes, including *vacuolar amino acid transporter 1* (*Solyc08g077823.1* and *Solyc09g098380.2*); *proline transporter 2* (*Solyc03g096380.3* and *Solyc05g052830.3*); *cationic amino acid transporter 8* (*Solyc12g011370. 2*); the *transcription of the uncharacterized protein LOC101268525* (*Solyc10g078470.2*), and downregulated the transcription of *cationic amino acid transporter 6* (*Solyc08g077823.1*) (Figure 6 and Table S2). This seems to be an adaptive response of tomato seedlings to LN levels, whereas Ni^2+^ stimulation restored the normal expression levels of the above amino acid transporter genes, suggesting that Ni^2+^ played a unique role in improving N utilization. On the other hand, N assimilation is a dynamic and complex process involving multiple genes [28]; in addition to NO_3_^−^ reduction, NH_4_^+^ assimilation, amino acid biosynthesis, and amino acid transport; it also includes NO_3_^−^ and NH_4_^+^ absorption and transportation. Studies have shown that a total of four protein families are involved in nitrate transport [29], of which the high-affinity nitrate transporter (NRT2) plays an important role in NO_3_^−^ uptake and N utilization. Normally, in the presence of a low N supply, NRT2 is activated to improve N uptake [30]. However, some reports suggest that both NRT2 and low-affinity nitrate transporters (PTR family/NRT1) contribute to meeting the plant’s nitrate demand [31,32]. In our study, *NRT1*, *NRT2*, and the chloride channel (CLC) protein family *CLC-b* responded positively to changes in N levels (Figure 6 and Table S2). Among them, *NRT2* (*Solyc00g090860.2* and *Solyc11g069750.2*) showed an obvious response to both low N and Ni^2+^ supply. Previous studies have shown that overexpression of *OsNRT2.3b* improves the uptake of nitrate in rice under low- and high-N supply conditions, thereby increasing yield [31]. Therefore, these candidate genes (*Solyc00g090860.2* and *Solyc11g069750.2*) may be the focus of future research to improve the uptake, transport, and utilization efficiency of NO_3_^−^ in the tomato seedling root system. Interestingly, under low-N conditions, the ammonium transporter *AMT1* (*Solyc09g090730.2*) and *AMT3* (*Solyc09g065740.2*) genes were upregulated in roots (Figure 6 and Table S2). Further, the AMT1 protein plays a key role in NH_4_^+^ uptake [33], suggesting that plants may employ alternative strategies (such as increased NH_4_^+^ uptake) to meet N requirements.

On the other hand, N availability affects C assimilation [34], and N assimilation depends on the C source and reducesthe power provided by C assimilation. This interaction has important implications for plant life activities. Generally, changes in environmental conditions lead to the disruption of metabolic balance in plants, as plants rely on the regulation of energy and material metabolism (e.g., C tuning and N assimilation) to adapt to stress [35]. In plants, sucrose (as the main nonstructural carbohydrate) can be hydrolyzedinto glucose and fructose, which in turn enter the EMP–TCA pathway to obtain a large amount of capacity and reduce the power required for the life activities of organisms. Under LN conditions, the contents of sucrose, glucose, and fructose in tomato roots were increased (Figure 1) and accompanied by an increase in the starch metabolism-related gene *α-Glucosidase* (*Solyc02g069670.3*) and the sucrose metabolism-related gene *INV* (*Solyc08g079080.4*). While the expression of those genes was upregulated, the expression of *SS* (*Solyc12g009300.3*) was downregulated (Figure 6 and Table S2). This means that the plant body is osmotically adjusted to maintain the osmotic pressure difference between the inside and outside of the cells. Furthermore, an LN supply inhibits energy metabolism, including the EMP–TCA pathway, in plants [36]. Our results also indicated that LN indeed suppressed the expression of the EMP-related genes *GAPDH* (*Solyc06g071920.3*), *PK* (*Solyc11g007690.2*), and *PDHB* (*Solyc06g072580.3*) (Figure 6 and Table S2). After Ni^2+^ application, the expression levels of related genes were restored, which may increase the availability of the C backbone for amino acid biosynthesis and other physiological and biochemical pathways and thus further promote the progression of related metabolic processes.

In summary, the application of Ni^2+^ as a regulatory strategy further leads to changes in metabolites and related metabolic pathways by altering the transcriptional levels and enzymatic activities of genes related to the C and N assimilation pathways (such as *NRT2*, *NR*/*NiR*, and *GS*/*GOGAT*). The results confirmed the effect of Ni^2+^ on C and N assimilation in tomato roots.

## 4. Materials and Methods

### 4.1. Plant Materials and Treatments

The experiment was carried out in the plant culture room of Fujian Agriculture and Forestry University. We used tomato (*Solanum lycopersicum* L. ‘Micro Tom’) seedlings grown in cavity trays withup to 5 leaves and 1 heart and then planted into hydroponic tanks and grown for 3 d. The experimental design is shown in Table 3 (for Ni^2+^ treatment, NiSO_4_ 6H_2_O was added to the hydroponic solution), and the specific nutrient solution configuration is shown in the Appendix A. The experiment was carried out at the end of 0 d, and the nutrient solution was changed every 3 d for a total of 3 times. The experiment was carried out with a completely randomized group design, consisting of four treatments.

### 4.2. Determination of Enzyme Activity in the C and N Metabolism

We used kits (from Comin, Suzhou, China) to determine the sucrose, glucose, fructose, starch, total amino acid, NO_3_^−^ and NH_4_^+^ contents; NR, NiR, GS, GOGAT, and GDH activities.

### 4.3. RNA-Seq and RT-qPCR Analysis

The transcriptomic data used in this experiment were provided by Beijing BioMarker and are detailed in the Appendix A. We screened for transcripts that met the criteria for |log_2_(fold change)| ≥ 1 and a *p*-adjusted value < 0.01 in regard to DEGs. For RT-qPCR analysis, total RNA was extracted from 0.3 g of root samples using the FastPure Plant Total RNA Isolation Kit (Polysaccharides & Polyphenolics-rich) (Vazyme, Nanjing, China). The kit used for reverse transcribing RNA and generating cDNA was FastKing RT SuperMix (Tiangen, Beijing, China), and the 2^−ΔΔCT^ method was used to calculate the relative expression levels of the genes. Please refer to Table 4 for the relevant gene sequence.

### 4.4. Statistical Analysis

We used DPS software 17.10 (Zhejiang University, Hangzhou, China) for statistical analysis. Additionally, Duncan’s multiple range test (*p* < 0.05) was used to analyzethe significant differences between experimental treatments.

## Figures and Tables

**Table 1:** Read quality in the RNA-sequencing (RNA-seq) analysis.

Sample Name	Clean Reads	Mapped Reads	Mapping Rate (%)	Uniq Mapped Reads	Uniq Mapping Rate (%)	Q30 (%)
NN_1	40,944,522	37,175,166	90.79	36,353,079	88.79	91.62
NN_2	42,183,292	36,617,683	86.81	35,772,363	84.80	91.17
NN_3	40,683,494	36,883,568	90.66	36,079,625	88.68	91.42
LN_1	54,418,492	49,722,687	91.37	48,893,527	89.85	91.45
LN_2	51,221,772	46,191,761	90.18	45,404,214	88.64	91.95
LN_3	44,687,904	38,817,524	86.86	38,152,833	85.38	91.91
Ni^2+^ + LN_1	40,918,376	37,467,413	91.57	36,774,788	89.87	91.85
Ni^2+^ + LN_2	43,462,968	39,298,045	90.42	38,608,365	88.83	91.85
Ni^2+^ + LN_3	41,466,278	37,055,020	89.36	36,343,722	87.65	91.76

**Table 2:** DEGs in the roots of tomato plants in response to Ni^2+^ and LN levels.

Gene	Gene ID	LN vs. NN		Ni^2+^ + LN vs. LN	
		RNA-Seq	RT-qPCR	RNA-Seq	RT-qPCR
*NR*	*Solyc11g013810.2*	−3.35	−0.08	4.32	1.40
*NiR*	*Solyc10g050890.2*	−3.57	−0.53	3.58	1.40
*GS*	*Solyc04g014510.3*	−2.43	−0.24	2.00	0.26
*GOGAT*	*Solyc03g083440.3*	−2.70	−1.36	2.54	0.35
*CA*	*Solyc09g010970.3*	1.08	0.34	−1.16	−0.92
*PK*	*Solyc11g007690.2*	−1.05	−0.10	1.15	0.58
*INV*	*Solyc08g079080.4*	3.09	1.72	−1.43	−1.17

Note: the number indicates log_2_(fold change).

**Table 3:** Design of experiment.

NiSO_4_ 6H_2_O Concentration (mg L^−1^)	N Concentration (mmol L^−1^)
0	0.383
	7.66
0.1	0.383
	7.66

**Table 4:** Primers used for RT-qPCR analysis.

Gene	Gene ID	Primer Sequences (Forward/Reverse)
*Actin*	*LOC101253675*	F 5′-GTCCTCTTCCAGCCATCCAT-3′ R 5′-ACCACTGAGCACAATGTTACCG-3′
*NR*	*Solyc11g013810.2*	F 5′-GCAACTTCCCTCCTTCATCCAACC-3′ R 5′-CGTCATCGTCATCCTCGTCTTCAC-3′
*NiR*	*Solyc10g050890.2*	F 5′-TGCTTGTGGGTGGATTCTTCAGTC-3′ R 5′-TTCTGCCTGTTCCCTCGGGTAC-3′
*GS*	*Solyc04g014510.3*	F 5′-CAACGGAGAAGTGATGCCTGGAC-3′ R 5′-GCCCACAACTCGTCACCTGATG-3′
*GOGAT*	*Solyc03g083440.3*	F 5′-GGCTGGTATGAGTGGTGGTGTTG-3′ R 5′-ACGCTGGTGTTGCTGTATCATCATC-3′
*CA*	*Solyc09g010970.3*	F 5′-TGGTGCCTCCTTATGGAGCTGATCC-3′ R 5′-CGAATGCCTCCACAGCGACTATG-3′
*PK*	*Solyc11g007690.2*	F 5′-TGCCTTGAATCGGGAATGTCTGTG-3′ R 5′-TAGTTCAGGACCACCAGTGTCTAGC-3′
*INV*	*Solyc08g079080.4*	F 5′-ACGGTAACAACGACGGTACTGATG-3′ R 5′-TCCTCATGGTGGTTAACGGCATTAG-3′

## Data Availability

Not applicable.

## Supplementary Materials

The following supporting information can be downloaded at: https://www.mdpi.com/article/10.3390/ijms231911398/s1.

## Appendix A

### Appendix A.1. N Nutrient Solution Formula

**Table A1:** N nutrient solution formula with different concentrations.

N Concentration (mmol L^−1^)	Reagent (mmol L^−1^)						
	Ca(NO_3_)_2_ 4H_2_O	CaCl_2_	KNO_3_	NH_4_H_2_PO_4_	KH_2_PO_4_	KCl	MgSO_4_ 7H_2_O
0.383	0.075	1.425	0.200	0.033	0.635	3.168	0.998
7.67	1.499		3.996	0.669			0.998

### Appendix A.2. RNA Extraction and RNA-Seq Analysis

We extracted the total RNA from 0.2 g roots samples using an RNAprep Pure Plant Kit (TIANGEN, Beijing, China). We then constructed and sequenced the mRNA libraries using an Illumina platform. After processing for quality control and data filtering, we mapped the clean reads to the reference genome(s) using TopHat2 (v2.2.13). We expressed the transcript level quantification as fragments per kilobase per million reads mapped. We performed a differ-ential expression analysis of the two groups using the DESeq R package (v1.10.1). We categorized the transcripts that satisfied the criteria |log_2_(fold change)| ≥ 1 and *p*-adjusted value < 0.01 as DEGs. We assigned the DEGs to obtain the GO annotations (i.e., functional classes of DEGs, including biological process, cellular component, and molecular function) to explore their involvement in biological pathways, and used KOBAS software to test the statistical enrichment of differentially expressed genes in KEGG pathways.
